# Draft Genome Sequence of *Burkholderia pseudomallei* Strain 350105, Isolated in Hainan, China, in 1976

**DOI:** 10.1128/genomeA.01162-15

**Published:** 2015-10-15

**Authors:** Lihua Song, Yonghui Yu, Le Feng, Jun He, Tao Wang, Hong Zhu, Qing Duan

**Affiliations:** State Key Laboratory of Pathogen and Biosecurity, Beijing Institute of Microbiology and Epidemiology, Beijing, China

## Abstract

*Burkholderia pseudomallei* is the etiological agent of the potentially fatal disease melioidosis. Here, we report the draft genome sequence of a virulent water isolate obtained from the Hainan Province of China in 1976, *B. pseudomallei* strain 350105.

## GENOME ANNOUNCEMENT

*Burkholderia pseudomallei* is a Gram-negative bacterium and the causative agent of melioidosis, a severe zoonosis that is mainly endemic in areas of Southeast Asia and northern Australia (1). Since its initial report in Hurma (Myanmar) in 1912 (2), it also been reported in Central America, the Caribbean, Africa, the Middle East, and China (3, 4). In China, melioidosis is primarily reported in the southeast coastal regions: Hainan, Guangxi, Guangdong, Fujian, Hong Kong, and Taiwan (5, 6). Many *B. pseudomallei* strains have been isolated from China and can be grouped into various biotypes and genotypes.

Here, we report the draft genome sequence of *B. pseudomallei* strain 350105, isolated from water in 1976 in Haikou city, Hainan Province, China. This strain can hydrolyze esculin and d-mannose but not l-arabinose, and it has a 50% lethal dose of <10 CFU by intraperitoneal infection of mice. Antimicrobial susceptibility tests revealed that it is resistant to multiple drugs, including cefepime, but it is susceptible to imipenem.

Strain 350105 was grown on Trypticase soy agar at 37°C for 24 h. Harvested bacteria were treated with protease K, allowing for DNA extraction using phenol-chloroform-isoamyl alcohol, followed by isopropanol precipitation, as previously described (7). The DNA was then used for full-length genome sequencing using a combination of sequencing platforms, specifically GS FLX 454 (Roche, Life Sciences) and Illumina GA (Life Technologies). GS FLX 454 sequencing results produced 677,973 reads, which were assembled with Newbler 2.3 to generate 758 contigs, with an average length of 9,263 bp (*N*_50_, 18,518 bp; total size, 7,021,855 bp). Two different DNA libraries (300-bp paired-end and 3,000-bp mate-pair) were sequenced using Illumina GA, yielding 34,300,804 and 23,487,886 reads, respectively. The mate-pair reads were scaffolded using SSPACE (8), producing 101 scaffolds with an average length of 73,956 bp and an *N*_50_ size of 432,106 bp. The assemblies and paired-end reads were postprocessed using GapCloser (9). The remaining gaps within the scaffolds were resolved using PCR and Sanger sequencing.

The draft genome of strain 350105 consists of two chromosomes totaling 7,127,033 bp in size. The G+C content is 64.4%. The open reading frames (ORFs) were analyzed with Glimmer 3.0 (10, 11), using a *Burkholderia* training set, predicting a total of 4,361 ORFs (>90 amino acids). The putative functions of these ORFs were annotated using BLASTp and the KEGG, COG, and GO protein databases (12, 13). Of the assembled sequence, 4.07% was identified as simple repeats using the RepBase17.07 database by RepeatMasker.

The genome sequence of strain 350105 will serve as a reference for additional assemblies of *B. pseudomallei* isolates from China. Moreover, future comparative genomic studies of isolates from both China and other areas will likely provide new insights into the molecular epidemiology of melioidosis.

### Nucleotide sequence accession numbers.

The draft genome sequences have been deposited in GenBank under the accession numbers CP012093 and CP012094.

## References

[1] Cheng AC, Currie BJ 2005 Melioidosis: epidemiology, pathophysiology, and management. Clin Microbiol Rev 18:383–416. doi:10.1128/CMR.18.2.383-416.2005. 15831829 PMC1082802

[2] Whitmore A, Krishnaswami CS 1912 An account of the discovery of a hitherto undescribed infective disease occurring among the population of Rangoon. Indian Med Gaz 47:262–267. PMC5168169 29005374

[3] Dance DA 2000 Melioidosis as an emerging global problem. Acta Trop 74:115–119. doi:10.1016/S0001-706X(99)00059-5. 10674638

[4] Currie BJ, Dance DA, Cheng AC 2008 The global distribution of *Burkholderia pseudomallei* and melioidosis: an update. Trans R Soc Trop Med Hyg 102(Suppl 1):S1–S4. doi:10.1016/S0035-9203(08)70002-6. 19121666

[5] Yang S 2000 Melioidosis research in China. Acta Trop 77:157–165. doi:10.1016/S0001-706X(00)00139-X. 11080506

[6] Li L, Lu Z, Han O 1994 Epidemiology of melioidosis in China. Zhonghua Liu Xing Bing Xue Za Zhi 15:292–295. (In Chinese.). 7532109

[7] Carlson JH, Porcella SF, McClarty G, Caldwell HD 2005 Comparative genomic analysis of *Chlamydia trachomatis* oculotropic and genitotropic strains. Infect Immun 73:6407–6418. doi:10.1128/IAI.73.10.6407-6418.2005. 16177312 PMC1230933

[8] Boetzer M, Henkel CV, Jansen HJ, Butler D, Pirovano W 2011 Scaffolding pre-assembled contigs using SSPACE. Bioinformatics 27:578–579. doi:10.1093/bioinformatics/btq683. 21149342

[9] Luo R, Liu B, Xie Y, Li Z, Huang W, Yuan J, He G, Chen Y, Pan Q, Liu Y, Tang J, Wu G, Zhang H, Shi Y, Liu Y, Yu C, Wang B, Lu Y, Han C, Cheung DW, Yiu SM, Peng S, Xiaoqian Z, Liu G, Liao X, Li Y, Yang H, Wang J, Lam TW, Wang J 2012 SOAP*denovo*2: an empirically improved memory-efficient short-read *de novo* assembler. GigaScience 1:18. doi:10.1186/2047-217X-1-18. 23587118 PMC3626529

[10] Delcher AL, Bratke KA, Powers EC, Salzberg SL 2007 Identifying bacterial genes and endosymbiont DNA with Glimmer. Bioinformatics 23:673–679. doi:10.1093/bioinformatics/btm009. 17237039 PMC2387122

[11] Delcher AL, Harmon D, Kasif S, White O, Salzberg SL 1999 Improved microbial gene identification with Glimmer. Nucleic Acids Res 27:4636–4641. doi:10.1093/nar/27.23.4636. 10556321 PMC148753

[12] Harris MA, Clark J, Ireland A, Lomax J, Ashburner M, Foulger R, Eilbeck K, Lewis S, Marshall B, Mungall C, Richter J, Rubin GM, Blake JA, Bult C, Dolan M, Drabkin H, Eppig JT, Hill DP, Ni L, Ringwald M, Balakrishnan R, Cherry JM, Christie KR, Costanzo MC, Dwight SS, Engel S, Fisk DG, Hirschman JE, Hong EL, Nash RS, Sethuraman A, Theesfeld CL, Botstein D, Dolinski K, Feierbach B, Berardini T, Mundodi S, Rhee SY, Apweiler R, Barrell D, Camon E, Dimmer E, Lee V, Chisholm R, Gaudet P, Kibbe W, Kishore R, Schwarz EM, Sternberg P, Gwinn M 2004 The Gene Ontology (GO) database and informatics resource. Nucleic Acids Res 32:D258–D261. doi:10.1093/nar/gkh036. 14681407 PMC308770

[13] Kanehisa M, Goto S 2000 KEGG: Kyoto Encyclopedia of Genes and Genomes. Nucleic Acids Res 28:27–30. doi:10.1093/nar/28.1.27. 10592173 PMC102409

